# Trophic Ecology of a Threatened Specialist: Implications of the Dependence on *Pappostipa frigida* for the Conservation of *Chinchilla chinchilla*

**DOI:** 10.3390/ani16010027

**Published:** 2025-12-22

**Authors:** Juan Pablo Castillo, Arturo Cortés, Fernando Novoa

**Affiliations:** 1 Laboratory of Animal Ecophysiology, Department of Biology, Universidad de La Serena, La Serena 1720256, Chile; 2 Center of Applied Ecology, La Reina 7850308, Santiago, Chile

**Keywords:** *Chinchilla chinchilla*, trophic ecology, diet selection, specialist, high-andean conservation

## Abstract

Understanding trophic ecology is fundamental for the conservation of threatened species with specialist trophic strategies, such as the short-tailed chinchilla (*Chinchilla chinchilla*), a critically endangered rodent whose diet in the wild is poorly understood. This study presents the first integrated annual characterization of the dietary habits, trophic niche, and resource selection patterns of a high-Andean population. Plant availability was assessed, and dietary composition was analyzed via seasonal microhistological analysis of fecal samples. Diversity (Shannon-Wiener, H′), overlap (Schoener, PS), and resource selection (Manly’s selection index) metrics were calculated. The results indicate a diet of very low diversity (H′ < 0.1), stable throughout the year (PS > 0.99), and dominated (>77%) by grass *Pappostipa frigida*, with significant positive selection in all seasons. Shrub species, such as *Adesmia frigida*, were consistently avoided. This high degree of specialization reflects low functional plasticity and highlights the high vulnerability of *C. chinchilla* to environmental changes and habitat loss, underscoring that the conservation and restoration of *P. frigida* grasslands are imperative for the species’ survival. Microhistological methodology is confirmed as a key tool for identifying critical trophic relationships and supporting conservation plans based on essential resources.

## Simple Summary

This study assesses the diet and trophic specialization of the short-tailed chinchilla (*Chinchilla chinchilla*), a critically endangered species from the Chilean Andes. The results show a strong dependence on a single plant resource (*Pappostipa frigida*), evidencing its vulnerability to changes in high-Andean vegetation and highlighting the need to conserve these grasslands.

## 1. Introduction

Trophic ecology is a fundamental pillar for understanding the autecology of a species and its role within an ecosystem [1]. This understanding is particularly crucial for herbivorous mammals inhabiting environments with limited and highly unpredictable resources, such as high-Andean ecosystems, where detailed knowledge regarding their diet, resource availability, and foraging strategies is essential for developing effective conservation plans [2]. The characterization of trophic parameters allows for the evaluation of a species’ degree of dependence on specific resources and its dietary flexibility in the face of environmental variations [3]. In high-Andean ecosystems, diverse herbivores, including camelids and rodents, present diets dominated by grasses, reflecting a specialized strategy within communities characterized by limited resources and extreme environmental conditions [4,5,6]. The evaluation of parameters such as dietary niche breadth, selectivity, and botanical composition of the diet constitutes an essential tool for understanding these feeding strategies and their relationship with seasonal forage availability [7].

In high-Andean ecosystems, distinct herbivores show trophic adaptations to forage availability and seasonality. The taruka (*Hippocamelus antisensis*) presents a primarily herbaceous diet, while the guanaco (*Lama guanicoe*) maintains a generalist consumption with a preference for grasses. In contrast, the Andean viscacha (*Lagidium viscacia*) exhibits a greater dependence on species of the genus *Stipa*, evidencing a trend towards specialization in arid high-altitude environments [8,9,10,11].

**Figure 1:**
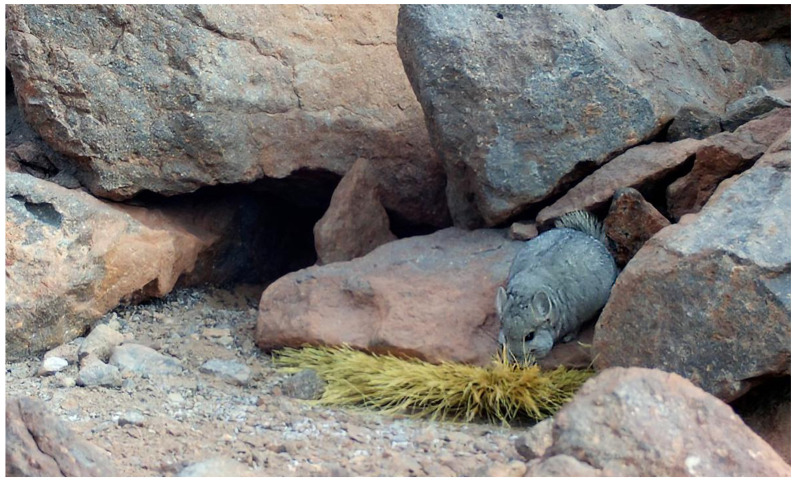
Individual short-tailed chinchilla (*Chinchilla chinchilla*) foraging in its natural high-Andean rocky habitat in the Atacama Region, Chile.

The short-tailed chinchilla (*Chinchilla chinchilla*) is a hystricomorph rodent endemic to the Andes, classified as Critically Endangered [12]. Historically, populations suffered a severe reduction due to overexploitation by the fur industry, driving them to the brink of extinction [13]. Currently, its study in natural conditions is limited due to the remote and extreme nature of its habitat—consisting of arid rocky outcrops between 3900 and 4900 m in altitude—as well as its crepuscular and nocturnal habits, which hinder direct observation (Figure 1) [14].

Despite its priority status for conservation, knowledge regarding the trophic ecology of *C. chinchilla* is notoriously fragmented. Most available information on the diet of *C. chinchilla* is limited primarily to qualitative descriptions of the plant species present in its habitat [15], without quantitative evaluations of composition or food selection. To date, only one study has quantified its diet through microhistological analysis, describing a consumption consisting mainly of grasses of the genus *Stipa* (currently *Pappostipa*) and, to a lesser extent, shrubs of the genus *Adesmia* [16]. However, that work was limited in its temporal scope, leaving a fundamental gap regarding the seasonal dynamics of the diet. It remains unknown whether *C. chinchilla* maintains a stable feeding regime throughout the year or if it exhibits trophic plasticity to adapt to seasonal variations in resource availability—an uncertainty that contrasts with the better understanding of its congener *C. lanigera*, for which data on dietary flexibility are available [6]. Furthermore, ecophysiological studies conducted on this species (under the synonym *C. brevicaudata*) have explicitly documented a reduced basal metabolism and high digestive retention capacity [17], key adaptations for processing fibrous diets in low-productivity environments.

Based on this specific physiological background and previous reports of grass consumption in high-Andean populations [16], it is hypothesized that the diet of *C. chinchilla* will be determined by a strategy of functional specialization. It is predicted that consumption will be concentrated mostly on the dominant grass resource (*Pappostipa frigida*) and that this pattern will remain stable seasonally through active selection, discriminating against the use of available shrub resources due to chemical restrictions (secondary metabolites) and physical constraints (e.g., thorns in *Adesmia*) that would limit energetic efficiency.

In this context, the objective of this study was to quantitatively characterize the trophic ecology of a population of *Chinchilla chinchilla* over a complete annual cycle in the Atacama region. Specifically, we proposed the following: (1) determine the composition, diversity, and stability of the diet across the four seasons; and (2) quantify resource selection patterns to identify the key plant species that sustain the population. This information is essential for generating an ecological baseline to support the development of evidence-based management and conservation strategies oriented toward this threatened species.

## 2. Materials and Methods

### 2.1. Study Area

**Figure 2:**
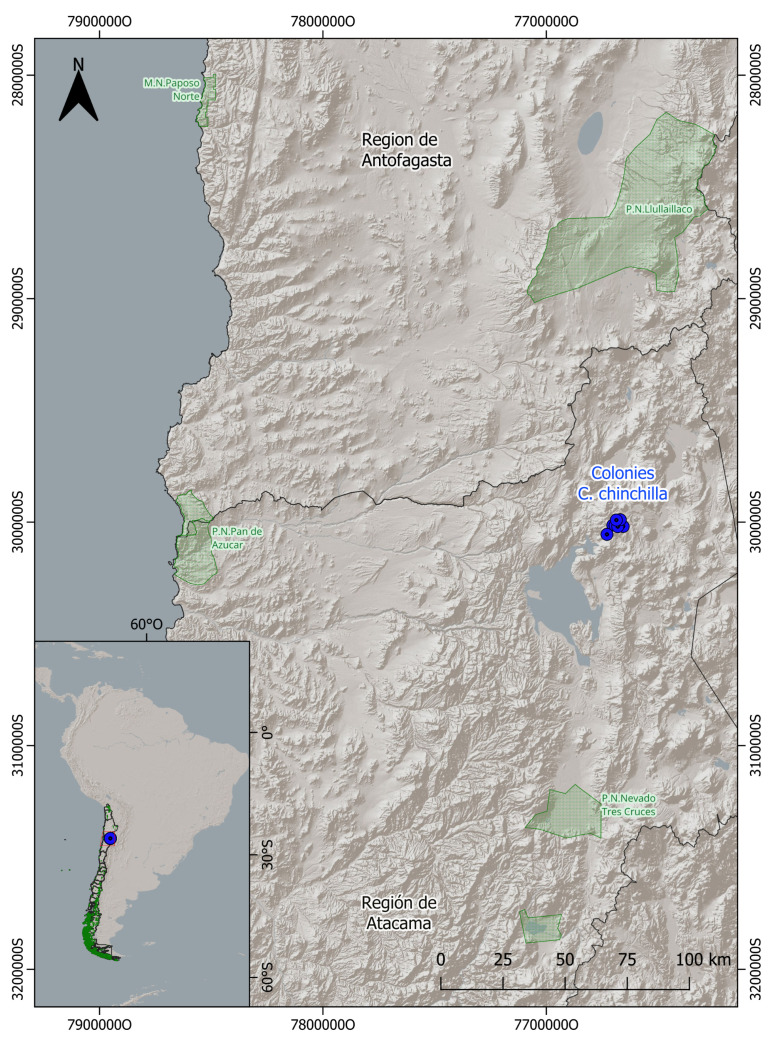
Study area in the Andes Mountains of the Atacama Region, northern Chile. Blue dots indicate the location of the 12 burrows (colonies) inhabited by *Chinchilla chinchilla* where sampling was conducted. These specific sites constitute the patches of effective habitat distributed within the general study matrix of approximately 1800 ha. The red box indicates the location of the enlarged study area shown in the main map. Nearby protected areas are shown in green polygons (P.N. = National Park, M.N. = Natural Monument) along with the site’s location in the context of South America (bottom left inset). Coordinate values correspond to UTM coordinates (meters), with “S” indicating the Southern Hemisphere and “O” indicating West (Oeste).

The study was conducted in the Atacama Region, Chañaral Province, in the commune of Diego de Almagro (90 km northeast of the city of the same name), covering an altitudinal range between 3900 and 4700 m a.s.l. (Figure 2). The general study area comprises approximately 1800 hectares and corresponds phytogeographically to the “Desert Steppe of the Andean Salt Flats” formation [18]. Climatically, the zone is classified as High Mountain Tundra, a regime combining desert and polar features characterized by low temperatures throughout the year, with an annual mean of −1.7 °C and a daily thermal oscillation exceeding 15 °C; the temperature difference between extreme months is nearly 10 °C, with a mean of 3.5 °C in January and −6.3 °C in July [19]. Annual precipitation is scarce (<150 mm) and presents a dual seasonal dynamic: snow events occur during the austral winter, while summer rains associated with the “Altiplanic Winter” phenomenon are recorded in summer [20]. The persistence of snow cover seasonally restricts access to trophic resources. In this context, the effective habitat of *C. chinchilla* is naturally fragmented, restricted to 12 rocky outcrops where the burrows (colonies) identified in this study are located, acting as islands of refuge and resource concentration within the arid matrix.

### 2.2. Trophic Availability and Diet Analysis

Sampling campaigns were carried out seasonally in Autumn (April 2017), Winter (August 2017), Spring (October 2017), and Summer (February 2018). Trophic resource availability was assessed by estimating seasonal vegetation cover using the point-intercept method [21]. Fifteen transects of 25 m in length were established, distributed proportionally among the studied rocky outcrops and placed randomly within the “feeding zones,” defined as the vegetated area located within a 200 m radius of active rocky shelters (a distance coinciding with the maximum displacement range observed for the species in the area). Cover was recorded every 25 cm, totaling 1500 points per season. Relative plant cover was used as a proxy for environmental availability.

Although fecal samples were successfully collected during all four seasons, direct vegetation measurement via transects was not feasible in winter due to the presence of snow cover. Consequently, winter availability was estimated using late autumn data as a valid biological proxy. This approach is supported by two lines of evidence: first, the phenological dynamics of the Puna indicate the cessation of vegetative growth by mid-May [20]; second, the dominance of *Pappostipa frigida* (>82% cover), a perennial grass that retains its senescent aerial biomass throughout the winter with minimal structural turnover until spring [18].

For dietary analysis, 12 fresh fecal samples were collected per season. This sample size (n=12 per season) was considered adequate based on methodological reviews indicating that, for major components or low-diversity diets, precision stabilizes with reduced sample sizes [22]. Given the specialist behavior of *C. chinchilla*, it is assumed that intra-seasonal dietary variability is lower than that of generalist herbivores, allowing for a faster saturation of the species accumulation curve. To ensure statistical independence and avoid pseudoreplication, samples were selected from active burrows located in distinct rocky outcrops or spatially separated by a minimum distance of 200 m, assuming they belonged to different individuals or family groups. Sixty histological slides were prepared per season (5 per sample) following the modified Williams technique [23]. Botanical composition was estimated by analyzing 10 microscopic fields per slide, selected systematically to avoid bias and overlap. Fragments lacking diagnostic epidermal characteristics were categorized as “unidentified fibers.”

### 2.3. Statistical Analysis

To quantitatively assess the trophic ecology of *Chinchilla chinchilla*, descriptive analyses of composition, niche, and selection were performed. To guarantee data independence and avoid pseudoreplication, fecal samples (n=12 per season) were collected from latrines or active crevices that were spatially separated (minimum distance >20 m), assuming they belonged to different individuals or family groups within the colony. All calculations and confidence interval estimations were performed using the Python programming language (version 3.11) and the scientific libraries NumPy (version 2.3.5) and Pandas (version 2.2.3) for data matrix manipulation.

#### 2.3.1. Trophic Niche Breadth and Diversity

The diversity of both environmental availability and diet for each season was calculated using the Shannon-Wiener index ($H^{\prime }$):

(1) $$H^{\prime }=-\sum_{i=1}^{S}p_{i}\ln p_{i}$$

where $p_{i}$ is the proportion of item *i* and *S* is the total richness of items [1]. To obtain robust estimates and assess statistical uncertainty, 95% confidence intervals (CI) for $H^{\prime }$ were generated using the non-parametric bootstrapping method. We performed 1000 iterations using multinomial resampling based on effective sample sizes reflecting the actual sampling effort: N=1500 simulated observations for vegetation availability (corresponding to total transect points) and N=600 for dietary use (corresponding to 60 slides × 10 microscopic fields).

#### 2.3.2. Estimation of Seasonal Trophic Overlap

To compare dietary similarity between different seasons, Schoener’s overlap index (PS) was used [1]:

(2) $$PS=1-0.5\sum_{i=1}^{n}\left|p_{xi}-p_{yi}\right|$$

where $p_{xi}$ and $p_{yi}$ are the proportions of item *i* in seasons *x* and *y*, respectively. A value of PS>0.60 was considered biologically significant.

#### 2.3.3. Estimation of Resource Selection

The selectivity of individual food items was determined by calculating Manly’s selection ratio ($w_{i}$) for Type II designs (individual animals identified, population-level availability) [24]:

(3) $$w_{i}=\frac{o_{i}}{\pi_{i}}$$

where $o_{i}$ is the proportion of resource *i* used (in the diet) and $\pi_{i}$ is the proportion of resource *i* available in the environment. For plant species present in the diet but with cover undetected in transects (i.e., relative availability = 0%), a nominal cover value of 1% was assigned to allow for coefficient calculation. To assign statistical significance to selection, 95% confidence intervals were estimated for each $w_{i}$ value via *bootstrapping* (1000 iterations) using the Bonferroni inequality. A species was considered positively selected (+) if the confidence interval lay entirely above 1, and negatively selected (−) if it lay entirely below 1.

## 3. Results

### 3.1. Resource Availability and Vegetation Cover

Total vegetation cover at the study site averaged 10.3% throughout the year, presenting marked seasonal variation. Absolute values fluctuated between a maximum of 15.18% during spring and a minimum of 1.52% in summer (Table 1). This summer minimum coincides with regional meteorological records for the summer of 2018, which documented an extreme precipitation deficit (0 mm accumulated between January and March at high-mountain reference stations) and temperatures above the historical average, creating a scenario of severe aridity that limited primary productivity [25].

Despite this environmental constraint, the structure of the plant community maintained its dominance: *Pappostipa frigida* invariably constituted more than 82% of the relative availability (proportional supply) across all seasons. In contrast, shrub species (e.g., *Adesmia frigida*, *Fabiana bryoides*) represented a minority fraction, with values consistently below 9% of relative cover (Table 1).

### 3.2. Diet Composition and Trophic Diversity

**Figure 3:**
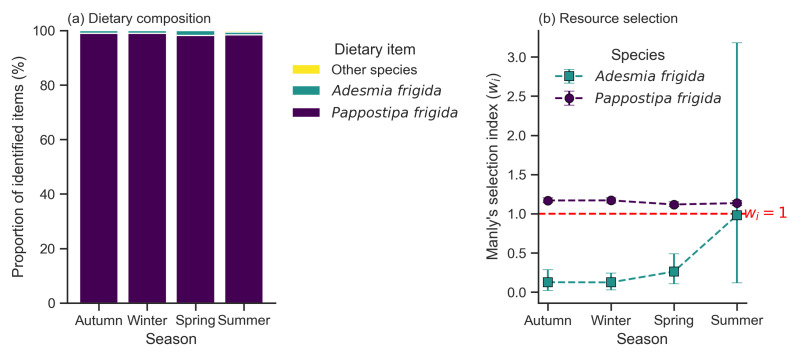
Trophic ecology of *Chinchilla chinchilla* across seasons. (**a**) Proportional dietary composition based on identified plant fragments. Percentages were normalized to 100% of the identified fraction for clarity. Unidentified material (fibers and other fragments) constituted approximately 20% of the total sample volume throughout the seasons and was excluded from this compositional analysis. The diet is consistently dominated by the grass *Pappostipa frigida*. (**b**) Resource selection analysis using Manly’s selection ratio ($w_{i}$) for the two main plant species. Values are shown with their 95% confidence intervals. The dashed line at $w_{i}=1$ indicates the threshold between selection and avoidance. *P. frigida* was consistently selected, while *Adesmia frigida* was consistently avoided. The wide confidence interval for *A. frigida* in summer reflects its sporadic and minimal consumption.

The diet of *Chinchilla chinchilla* was composed of five plant species. *Pappostipa frigida* was the principal dietary component in all seasons, with a contribution varying from a minimum of 77.58% in spring to a maximum of 82.36% in summer (Table 2; Figure 3a). *Adesmia frigida* was the second most consumed item, although its contribution did not exceed 1.44%. Species available in the habitat such as *Fabiana bryoides*, *Senecio rahmeri*, and *Menonvillea cuneata* were not detected in the diet.

Diversity analysis reflected the conditions of an ecosystem with low species richness. The diversity of vegetation availability ($H^{\prime }$ Availability) showed consistently low values, fluctuating between $H^{\prime }=0.4536$ in spring and $H^{\prime }=0.5970$ in summer. Even more notably, dietary diversity ($H^{\prime }$ Diet) was markedly lower, with values ranging between $H^{\prime }=0.057$ and $H^{\prime }=0.096$. This difference between environmental and consumer diversity underscores the high degree of trophic specialization of the species (Table 3).

### 3.3. Seasonal Trophic Overlap

The analysis of dietary overlap between seasons yielded high values, with Schoener’s index (PS) exceeding 0.99 in all pairwise comparisons (Table 3). These results indicate that the diet composition of *C. chinchilla* was practically identical and showed no biologically significant variations throughout the year.

### 3.4. Resource Selection

Resource selection analysis indicated non-random consumption patterns (Table 4). The main species in the diet, *Pappostipa frigida*, was consumed in a proportion greater than its availability, showing significant positive selection (+) in all four seasons (Figure 3b).

For *Adesmia frigida*, a seasonal pattern was observed: it was significantly avoided (−) during autumn, winter, and spring. In summer, however, its consumption was proportional to its availability, showing no significant selection (ns).

The shrub species *Fabiana bryoides* and *Senecio rahmeri*, along with the herbs *Menonvillea cuneata*, *Baccharis tola*, and *Oxalis pycnophylla*, were negatively selected (−). Finally, *Cistanthe minuscula* was consumed in proportion to its availability during summer, with no significant selection (ns) (Table 4).

## 4. Discussion

The trophic ecology of *Chinchilla chinchilla*, a species listed as Critically Endangered, has remained largely unexplored, in marked contrast to the knowledge available for its congener *C. lanigera*, characterized as a generalist and opportunistic herbivore [6,15,26]. The foraging strategy of *C. chinchilla* had been inferred primarily from a single quantitative study [16]. Our research provides the first detailed seasonal assessment of the diet and food selection of *C. chinchilla* in a high-Andean ecosystem, offering robust evidence to characterize its trophic strategy.

The results reveal unequivocal specialist behavior, defined by a diet of very low diversity, stable throughout the year, and based on the positive selection of a single grass, *Pappostipa frigida*. This dependence highlights the ecological relevance of this plant resource for the rodent’s survival and evidences a specialized strategy in the face of a low-productivity environment.

### 4.1. Trophic Strategy: A Specialist in a Low-Diversity Environment

This pattern contrasts strongly with *C. lanigera*, described as generalist and opportunistic [6,15,26], and validates our working hypothesis regarding niche differentiation between the two species, while *C. lanigera* is restricted to lower-altitude coastal and pre-Andean ranges characterized by milder climates and a diverse and seasonally variable plant supply [13,15], *C. chinchilla* inhabits a Puna ecosystem where floristic diversity is low and the availability of quality resources is restrictive. This differential environmental pressure likely forced an evolutionary divergence: the heterogeneity of the coastal scrubland favored dietary plasticity in *C. lanigera*, whereas the homogeneity and harshness of the high-Andean steppe directed *C. chinchilla* toward specialization in the most predictable and abundant resource (*P. frigida*), sacrificing niche breadth in exchange for greater efficiency in fiber processing.

Evidence of active and positive selection of *P. frigida*, rather than passive consumption, reinforces the specialist character of *C. chinchilla* in this demanding environment. This specialization appears to be sustained by physiological adaptations described for the genus, such as the capacity to process diets high in fiber and low in nutrients [17], which is crucial in high-Andean steppes [2]. This pattern coincides with other high-altitude Andean mammals (e.g., *Abrocoma cinerea*, *Lagidium viscacia*) whose diets, dominated by Poaceae and exhibiting low trophic plasticity, have been documented via microhistology [10]. Taken together, the results suggest a shared physiological limitation regarding lignified shrubs, orienting selection toward grasses with high fiber content.

This physiological efficiency is fundamental for interpreting the species’ response to extreme environmental fluctuations. Although the literature describes summer as a season of vegetative growth in the Puna [27], the minimum cover recorded in our study (1.52%) reflects the severe drought conditions that affected the region during the summer of 2018, characterized by a lack of precipitation and heatwaves [25]. Faced with this abiotic restriction preventing green regrowth, the diet of *C. chinchilla* remained unaltered. This confirms that the species is capable of decoupling itself from phenology, validating a resilient short-term specialization strategy.

However, this obligate functional dependence on a single resource carries inherent risks. Concordantly, recent studies warn that the resilience of specialist mammals is critically linked to the stability of their key trophic resources; therefore, significant or chronic alterations in *P. frigida* populations could exceed the ecological resilience thresholds of these colonies [28].

### 4.2. Resource Selection and Foraging Strategy

The constant positive selection of *P. frigida* establishes this species as a key resource. The avoidance of resources with chemical and physical defenses also influences the strategy: *Adesmia frigida* was avoided in three seasons, and shrubs such as *Fabiana bryoides*—whose congeneric species, like *F. patagonica*, present secondary metabolites with diuretic effects [29]—were not recorded in the diet. The seasonal variation in *A. frigida* (not significant in summer) could be related to greater leaf biomass during the growing season, reducing the efficacy of physical defenses and allowing incidental consumption. Even rare herbs consumed exclusively in summer (*Baccharis tola*, *Oxalis pycnophylla*) showed negative selection, suggesting incidental events rather than active diversification, which reinforces low trophic plasticity.

### 4.3. Trophic Context and Relationships with Other Herbivores

The documented patterns are consistent with regional work. Tirado et al. [16] reported dominance of *Stipa* (=*Pappostipa*) and secondary consumption of *Adesmia*. This specialization and avoidance of shrubs may reduce competition with other Andean rodents, such as *Abrocoma cinerea*, a consumer of resinous shrubs [10]. Such interactions are key to understanding the high-Andean community assembly and should be incorporated into conservation strategies.

### 4.4. Global Perspective and Methodological Approach

Trophic specialization is a frequent strategy in extreme ecosystems but increases vulnerability to environmental changes and loss of key resources [30]. Paleontological evidence indicates that the disappearance of specific food sources has been a determinant in the extinctions of specialists [31].

Methodologically, microhistological analysis is confirmed as a robust and cost-effective tool for cryptic or nocturnal species [7,23,32,33,34], allowing for the contrast of availability and use via indices such as Manly’s, while this technique presents inherent limitations, such as the presence of an unidentified material fraction (ca. 20% in this study), this is attributable to differential digestion and the loss of epidermal characteristics in highly lignified tissues. Nevertheless, it is unlikely that this fraction biases the conclusion of specialization: given the fibrous nature and dominance of *P. frigida*, it is reasonable to infer that a large part of these fibers corresponds to vascular tissue of this same species that lost its diagnostic features. Even assuming a conservative scenario, the high proportion of *P. frigida* in the identified fraction ratifies the specialist strategy.

To overcome these resolution limitations in the future, integration with DNA metabarcoding and automated classification (deep learning) offers increased taxonomic precision and efficiency [34,35], a relevant projection for non-invasive monitoring of *C. chinchilla*.

## 5. Conclusions

This study concludes that *Chinchilla chinchilla*, in the high-Andean ecosystem of Atacama, is an herbivore with a markedly specialist trophic strategy, rather than a generalist opportunist. The diet is stable throughout the year, based on positive selection and high dependence on the dominant grass *Pappostipa frigida*, with systematic avoidance of the available shrub flora.

These findings fill a fundamental gap in the autecology of this critically endangered species, providing the first quantitative evidence of its seasonal trophic niche and feeding behavior. The unique dependence on *P. frigida* makes the viability of populations inseparable from the conservation of these grasslands; protecting the plant resource is as essential as safeguarding the colonies.

Furthermore, this case illustrates the need for detailed studies and robust methodologies to move from generalist approaches to specific, evidence-based plans. In line with global evidence, reliance on few food resources is associated with a higher extinction risk in mammals [28,31]; specialization reduces resilience to environmental alteration [36]. Finally, these results acquire critical relevance in the face of climate change scenarios projecting increased aridification in the Puna. The obligate dependence of *C. chinchilla* on *P. frigida* suggests that monitoring the health of these tussock grasslands may serve as an early warning indicator for population viability, anticipating potential local collapses in the face of the degradation of its single key resource. Integrating trophic indicators into risk assessments and conservation planning is, therefore, a priority.

## Figures and Tables

**Table 1:** Absolute (A%) and relative (R%) vegetation cover in the study area for each season. Winter cover was estimated using autumn data as a proxy due to snow presence.

	Autumn 2017		Winter 2017		Spring 2017		Summer 2018	
Species	A%	R%	A%	R%	A%	R%	A%	R%
**SHRUBS**								
*Baccharis tola*	0.00	0.00	0.00	0.00	0.00	0.00	0.00	0.00
*Senecio rahmeri*	0.00	0.00	0.00	0.00	0.00	0.00	0.07	4.35
*Fabiana bryoides*	0.73	7.05	0.73	7.05	0.79	5.22	0.13	8.70
*Adesmia frigida*	0.86	8.33	0.86	8.33	1.06	6.96	0.00	0.00
**HERBS**								
*Menonvillea cuneata*	0.00	0.00	0.00	0.00	0.00	0.00	0.07	4.35
*Pappostipa frigida*	8.71	84.62	8.71	84.62	13.33	87.83	1.25	82.60
*Cistanthe minuscula*	0.00	0.00	0.00	0.00	0.00	0.00	0.00	0.00
*Oxalis pycnophylla*	0.00	0.00	0.00	0.00	0.00	0.00	0.00	0.00
Bare Soil	89.70	–	89.70	–	84.82	–	98.48	–

**Table 2:** Seasonal comparison between relative availability of plant resources (%) and their utilization in the diet of *Chinchilla chinchilla* (%).

	Autumn 2017		Winter 2017		Spring 2017		Summer 2018	
Dietary Item	Available %	Used %	Available %	Used %	Available %	Used %	Available %	Used %
*Pappostipa frigida*	84.62	78.37	84.62	77.66	87.83	77.58	82.60	82.36
*Adesmia frigida*	8.33	0.83	8.33	0.81	6.96	1.44	0.00	0.86
*Cistanthe minuscula*	0.00	0.00	0.00	0.00	0.00	0.00	0.00	0.40
*Baccharis tola*	0.00	0.00	0.00	0.00	0.00	0.00	0.00	0.04
*Oxalis pycnophylla*	0.00	0.00	0.00	0.00	0.00	0.00	0.00	0.04
*Fabiana bryoides*	7.05	0.00	7.05	0.00	5.22	0.00	8.70	0.00
*Senecio rahmeri*	0.00	0.00	0.00	0.00	0.00	0.00	4.35	0.00
*Menonvillea cuneata*	0.00	0.00	0.00	0.00	0.00	0.00	4.35	0.00
Unidentified fibers	–	20.35	–	19.95	–	17.90	–	16.30
Unrecognized material	–	0.45	–	1.58	–	3.08	–	0.00

**Table 3:** Trophic diversity and diet overlap indices for *Chinchilla chinchilla* by season.

Index and Comparison	Value	95% CI
**Shannon–Wiener Index ($H^{\prime }$)**		
*H′ Availability*		
Autumn 2017	0.5353	(0.4884–0.5777)
Winter 2017	0.5353	(0.4882–0.5750)
Spring 2017	0.4536	(0.4075–0.4965)
Summer 2018	0.5970	(0.5345–0.6522)
*H′ Diet*		
Autumn 2017	0.0583	(0.0223–0.0980)
Winter 2017	0.0574	(0.0223–0.0980)
Spring 2017	0.0909	(0.0482–0.1347)
Summer 2018	0.0963	(0.0438–0.1470)
**Schoener’s Overlap Index (PS)**		
Autumn vs. Winter	0.9998	(0.9867–1.0000)
Autumn vs. Spring	0.9923	(0.9783–1.0000)
Autumn vs. Summer	0.9942	(0.9800–0.9983)
Winter vs. Spring	0.9921	(0.9783–1.0000)
Winter vs. Summer	0.9942	(0.9800–0.9983)
Spring vs. Summer	0.9921	(0.9767–0.9967)

**Table 4:** Resource selection by *Chinchilla chinchilla* in each season. The table shows the used proportion ($p_{u}$), available proportion ($p_{d}$), Manly’s selection ratio ($w_{i}$) with its 95% confidence interval (CI), and the selection result: (+) positive selection, (−) negative selection, (ns) not significant.

Species	Season	$p_{u}$	$p_{d}$	$w_{i}$ (95% CI)	Selection
*Pappostipa frigida*	Autumn	0.9895	0.8462	1.169 (1.140–1.204)	(+)
	Winter	0.9897	0.8462	1.170 (1.137–1.204)	(+)
	Spring	0.9818	0.8783	1.118 (1.089–1.150)	(+)
	Summer	0.9840	0.8260	1.135 (1.104–1.168)	(+)
*Adesmia frigida*	Autumn	0.0105	0.0833	0.126 (0.019–0.285)	(−)
	Winter	0.0103	0.0833	0.124 (0.028–0.243)	(−)
	Spring	0.0182	0.0696	0.262 (0.106–0.489)	(−)
	Summer	0.0103	0.0100 ^a^	0.981 (0.116–3.179)	(ns)
*Fabiana bryoides*	Autumn	0.0000	0.0705	0.000	(−)
	Winter	0.0000	0.0705	0.000	(−)
	Spring	0.0000	0.0522	0.000	(−)
*Baccharis tola*	Summer	0.0005	0.0100 ^a^	0.048 (0.000–0.433)	(−)
*Cistanthe minuscula*	Summer	0.0048	0.0100 ^a^	0.457 (0.000–1.718)	(ns)
*Oxalis pycnophylla*	Summer	0.0005	0.0100 ^a^	0.048 (0.000–0.474)	(−)
*Menonvillea cuneata*	Summer	0.0000	0.0435	0.000	(−)
*Senecio rahmeri*	Summer	0.0000	0.0435	0.000	(−)

^a^ A nominal availability value of 1% was assigned to species present in the diet but not detected in transects to allow for the calculation of the selection index.

## Data Availability

The data supporting the results are available upon reasonable request from the corresponding author.
